# Successful treatment of facial telangiectasia from carcinoid syndrome with pulsed dye laser therapy

**DOI:** 10.1016/j.jdcr.2023.08.022

**Published:** 2023-08-30

**Authors:** Roy Jiang, Jonathan Leventhal, Kathleen C. Suozzi

**Affiliations:** Department of Dermatology, Yale School of Medicine, New Haven, Connecticut

**Keywords:** carcinoid syndrome, facial telangiectasia, flushing, pulsed dye laser, rosacea

## Introduction

Carcinoid syndrome is a common sequela of liver metastatic neuroendocrine tumors, arising from the production of vasodilatory amines by the tumor which bypasses liver degradation.^1^^,^^2^ Rosacea-like changes with telangiectasias commonly result from recurrent facial flushing due to carcinoid syndrome. Flushing from carcinoid syndrome is episodic, resulting in salmon pink to red discoloration depending on characteristics of the tumor.^3^ No treatments for rosacea-like changes with telangiectasias from carcinoid syndrome associated flushing are reported in the literature. We report a case of successful treatment of rosacea-like changes with telangiectasias with pulsed dye laser (PDL) in a 57-year-old patient who developed carcinoid syndrome from neuro-endocrine tumor metastases.

## Case presentation

A 57-year-old female presented with symptoms of abdominal pain, bloating, urinary urgency and weight loss. The patient also experienced diarrhea and flushing multiple times a week. Imaging revealed lesions in the right pelvis, mesentery, diffuse adenopathy and a lesion in the right hepatic lobe. Histopathologic features from a lymph node biopsy were consistent with a low-grade neuroendocrine tumor. Octreoscan showed increased uptake in the large pelvic mass, nodular implants, mesenteric and retroperitoneal adenopathy, the C1 vertebral body, and the right hepatic lobe lesion suggestive of metastatic disease. An initial chromogranin A level of 230 ng/L was noted and tricuspid regurgitation was seen on echocardiography. These findings were most consistent with carcinoid syndrome, and the patient was started on somatostatin analog (SSA, sandostatin 30 mg intramuscular) injections monthly. Other treatments that the patient received included palliative radiation to the C1 vertebra, and peptide receptor radionuclide therapy (PRRT, ^177^Lu-Dotatate).

**Fig 1:**
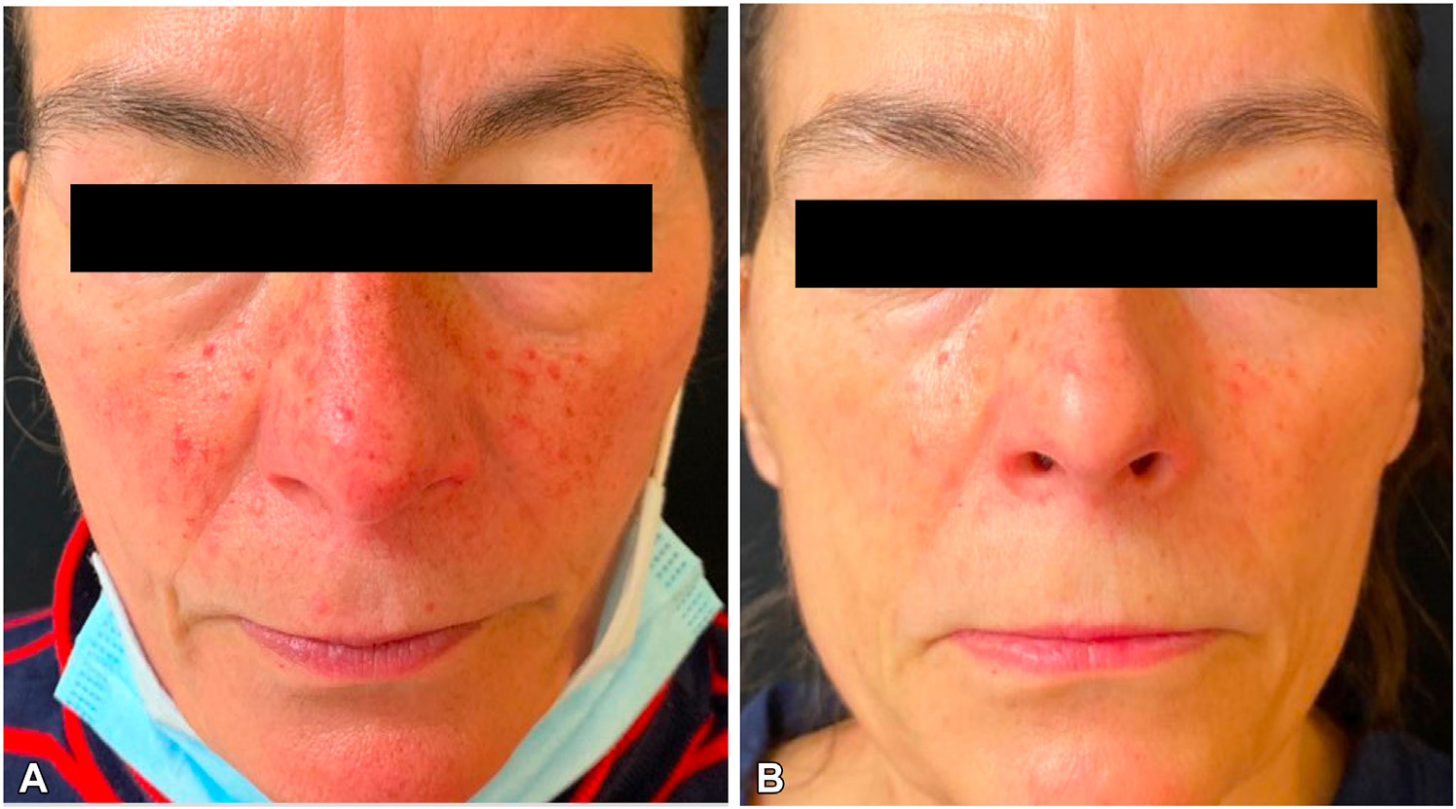
**A,** Frontal view of face demonstrating cutaneous findings consistent with rosacea-like changes with telangiectasias prior to therapy. **B,** Frontal view of face after completion of 8 sessions of treatment, 8 months later.

At the time of diagnosis, the patient reported episodes of bright red flushing lasting up to 10-15 mins. She stated that she had a several year history of sporadic flushing episodes triggered by heat or exercise prior to her diagnosis but no personal or family history of rosacea. The patient reported that SSA treatment improved her diarrhea but not her flushing. Rosacea-like changes with telangiectasias developed over the same distribution as her flushing. Five years after the patient was diagnosed with carcinoid syndrome, the patient presented to our clinic with widespread facial telangiectasia and spider angiomas over the chin, nose, and the cheeks in a malar distribution (Fig 1).

The patient was started on a trial of PDL treatment and received 6 treatments using 595 nm PDL (VBeam Perfecta, Candela). Treatments were scheduled every month and involved 3-10 ms pulses with a 7-10 mm round spot or a 10 × 3 mm rectangular spot for discrete telangiectasias with an average of approximately 100 pulses per session. Energy range of 6-7 mJ with the round spot sizes and 12 mJ with the rectangular spot size were used (see Table I). Facial telangiectasia became significantly diminished with improvement noted after the first session. After 6 treatment sessions, the patient presented with numerous pustules, many overlying her angioma-like telangiectasias in the central face over medial cheeks and nose. She had not previously had a papulopustular flare. She was started on topical ivermectin with resolution of the papulopustular flare. She continued to improve with continuation of regular sessions with PDL and now has minimal facial telangiectasias.

**Table I:** Parameters for 8 PDL laser treatment sessions are provided. Here, both 3 × 10 mm rectangular or 7 mm or 10 mm round spot sizes were used. Parameters for these 2 spot sizes are provided for each session if both were used

Session number	Wavelength (nm)	Fluence (J/cm2)	Pulse durations (ms)	Spot size (mm)	Pulses (count)
1	595	12.5	10	3 × 10	100
2	595	13, 6.5	10, 3	3 × 10, 10	47, 72
3	595	12.5, 6.5	10, 3	3 × 10, 10	27, 93
4	595	9	3	7	53
5	595	10, 6	3, 3	7, 10	19, 72
6	595	10, 6	3, 3	7, 10	17, 80
7	595	6	3	10	98
8	595	6	3	10	69

## Discussion

Overall, 10% to 20% of neuroendocrine tumor diagnoses with metastases to the abdomen may present with carcinoid syndrome, and flushing is the most common symptom.^1^^,^^3^^,^^4^ Several trials have shown that SAA treatment is sufficient for reducing flushing episodes from carcinoid syndrome.^4^^,^^5^ A systematic review noted that first line use of SSAs may reduce flushing in 69% to 72% of participants, while PRRT may reduce flushing in 56% of participants.^5^^,^^6^ Complete resolution of flushing is rare, although cases have been reported following SSA treatment or resection of the primary/secondary tumor.^3^

There are currently no treatment options for rosacea-like changes with telangiectasias from chronic carcinoid syndrome. However, PDL treatment has been shown to be effective for the treatment of facial telangiectasia as a result of rosacea, age, or photo-damage.^7^ PDL treatments target and shrink blood vessels deep in the skin, reducing their surface appearance; they are an effective treatment for facial telangiectasia and rosacea in terms of improved quality of life and appearance.^8^

Prolonged vasodilation from flushing secondary to carcinoid syndrome may lead to the development of rosacea-like changes with telangiectasias. In 1 study by Bell et al^3^, 24 of 25 patients with carcinoid syndrome developed flushing, 3 of whom eventually developed facial telangiectasias and/or rosacea-like changes. Patients were only diagnosed after several years of recurrent flushing as a result of delays in diagnosis. It is notable that our patient reported a history in sporadic flushing prior to her carcinoid diagnosis. While delays in diagnosis have not been shown to correlate with survival or disease extent, the mean delay for carcinoid tumor diagnosis is 66 months; initial mis-diagnosis of carcinoid syndrome as undifferentiated rosacea and flushing leads to delays in diagnosis of 200 months or 99 months respectively.^9^ Thus, patients may have a prolonged history of flushing prior to definitive diagnosis.

This case highlights how rosacea-like changes progress with time; the march from flushing to telangiectasias to papulopustular flares can take years. It is important to consider additional medical therapy, such as ivermectin 2% cream in this case, to mitigate papulopustular flares. Studies on the use of PDL for rosacea-like changes arising secondary to other neuroendocrine conditions and conditions associated with recurrent flushing (mastocytosis, medullary thyroid cancer, pheochromocytoma, and endogenous Cushing's syndrome) would represent an important future direction to pursue.^10^ In addition, the pathogenesis of rosacea development in carcinoid patients underscores the importance of the vascular prominence in the disease process and supports the notion that early intervention with PDL, or other vascular lasers such as 532 nm KTP or intense pulsed light (IPL), in not just carcinoid patients but in traditional rosacea patients can mitigate disease progression and may decrease the risk for development of papulopustular flares while providing symptomatic relief to flushing.

## Conflicts of interest

None disclosed.
